# Experimental Investigation of a Waste-Derived Biopolymer for Enhanced Oil Recovery Under Harsh Conditions: Extraction and Performance Evaluation

**DOI:** 10.3390/polym17212896

**Published:** 2025-10-30

**Authors:** Ammar G. Ali, Faisal S. Altawati, Osama A. Elmahdy, Fahd M. Alqahtani, Mohammed T. Althehibey, Taha M. Moawad

**Affiliations:** Department of Petroleum and Natural Gas Engineering, College of Engineering, King Saud University, Riyadh P.O. Box 800, Saudi Arabiatmoawad@ksu.edu.sa (T.M.M.)

**Keywords:** enhanced oil recovery (EOR), core-flooding, polymer flooding, biopolymer, pomegranate peel, waste material, environmental-friendly solutions

## Abstract

Aligned with Saudi Arabia’s Vision 2030 and its corresponding global direction, this study aimed to identify and evaluate an environmentally friendly and alternative material to replace conventional synthetic polymers for polymer flooding. Extracting biopolymer solution, characterizing rheological properties, and conducting core-flooding experiments (seawater flood (SWF), secondary polymer flood (PF), and tertiary polymer flood) were experimentally investigated under simulated reservoir conditions (75 °C, 165,000 ppm TDS brine, and 2000 psi pore pressure). Biopolymer solutions were successfully generated from powdered pomegranate peels, and rheological characterizations of solutions with different shear rates, temperatures, and pomegranate-peel concentrations were investigated. Results revealed that significant shear-thinning behavior was pronounced in the biopolymer solutions, where 7% solution was selected for core-flooding tests. 7% solution exhibited 14.4 cP apparent viscosity at 13.2 s^−1^ shear rate and 75 °C, indicating good thermal stability. Interfacial tension (IFT) results demonstrated high IFTs compared to the required IFT to reduce capillary forces, indicating that improved mobility control through viscosity enhancement serves as dominant EOR mechanism. The results indicated that PF yielded a higher ultimate oil recovery (62.2%) compared to SWF (47.6%) and tertiary polymer flood (58.0%). Results demonstrated that significant pressure fluctuations during polymer injection were observed, highlighting injectivity challenges. From all results, pomegranate peels would be potentially used to generate a biopolymer solution and replace environmentally hazardous materials.

## 1. Introduction

Global energy consumption continues to rely heavily on fossil fuels, which collectively account for 81.3% of total energy supply, with oil leading at 196 exajoules (EJ) (31.7%), followed by coal at 164 EJ (26.5%), and natural gas at 144 EJ (23.2%) [1]. In 2024, global energy demand increased to nearly 650 EJ (2.2%), with renewables accounting for 38% of supply growth, compared to oil’s 11% contribution [2]. As a result, the oil demand is projected to peak around 2029–2030 at approximately 103 million barrels per day (mb/d) before a gradual decline, creating urgency for maximizing recovery from existing reserves during this critical transition period.

This sustained fossil fuel dominance, particularly oil’s continued prominence, faces dual pressures from declining production in mature fields and evolving market dynamics driven by the energy transition. Conventional primary and secondary recovery methods typically leave 50–70% of OOIP unrecovered in mature reservoirs, representing a vast untapped hydrocarbon resource that EOR techniques can mobilize [3,4,5]. EOR methods, which significantly contribute to global oil production, employ advanced tertiary recovery technologies to extend the productive lifespan of reservoirs and optimize the utilization of existing petroleum assets. Among chemical EOR processes, polymer flooding has proven particularly successful due to its ability to improve sweep efficiency and ultimate recovery [6,7]. Therefore, considerable attention is being directed toward the development and optimization of novel green biopolymers as eco-friendly alternatives while supporting the petroleum industry’s sustainability objectives. Nevertheless, critical challenges remain in developing such environmentally sustainable polymer solutions.

Among various EOR methods, chemical EOR represents a significant advancement in petroleum extraction technology. Chemical EOR aims to alter fluid or rock-fluid properties to mobilize trapped oil [8,9]. Polymer flooding stands as a mature and widely implemented chemical EOR technology globally, with its effectiveness stemming from straightforward application and proven field results. Polymer flooding enhances oil recovery through multiple synergistic mechanisms operating at different scales. While improved macroscopic sweep efficiency represents the primary mechanism, microscopic displacement effects and other phenomena contribute significantly to overall recovery performance.

**Figure 1:**
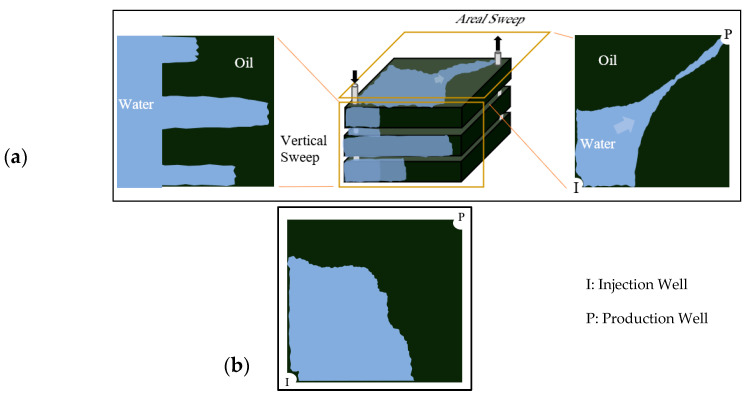
(**a**) areal and vertical sweep for water flooding, (**b**) improved sweep efficiency achieved with polymer flooding. Reproduced with permission from [8], Springer Nature, 2024.

The fundamental principle of polymer flooding involves adding water-soluble polymers to the injection water, thereby substantially increasing its viscosity [10,11]. This modification creates a more favorable mobility ratio (M) between displaced and displacing fluids [12,13]. A lower mobility ratio stabilizes the displacement front and suppresses viscous fingering phenomena, where low-viscosity water preferentially bypasses oil through high-permeability pathways [7,14,15,16] as illustrated in Figure 1a. Consequently, the injected fluid contacts and sweeps a larger reservoir volume, recovering oil previously bypassed by conventional waterflooding (Figure 1b).

Specific polymers exhibit Disproportionate Permeability Reduction (DPR), preferentially reducing water relative permeability ($k_{rw}$) more than oil relative permeability ($k_{ro}$) [6,7]. This phenomenon occurs through polymer adsorption, mechanical entrapment, or selective layer formation within porous media [5,17,18]. DPR improves water-injection profiles by diverting fluids from high-permeability “thief” zones, thereby enhancing both vertical and areal sweep efficiencies [19]. Additionally, polymer retention through adsorption and mechanical entrapment reduces permeability in swept zones even after polymer injection ceases, improving post-polymer waterflooding. Subsequent water would follow paths of higher resistance, leading to more uniform sweep patterns and continued oil mobilization. This effect can also reduce water cut earlier in a project’s lifecycle, decreasing water handling requirements and operational costs [7]. Beyond traditional macroscopic sweep improvement, specific synthetic polymers exhibit viscoelasticity under reservoir conditions [20,21]. This property generates forces capable of mobilizing residual oil trapped by capillary action due to polymer chain stretching in extensional flow fields within converging-diverging pore throats [22]. Mechanisms such as pulling, stripping, and oil-thread mobilization can reduce residual oil saturation ($S_{or}$) potentially [21,23,24]. However, quantification of this effect under diverse reservoir conditions remains an active area of research [25].

Synthetic polymers have historically dominated commercial EOR applications due to their cost-effectiveness and well-established performance characteristics. Partially Hydrolyzed Polyacrylamide (HPAM) represents the most widely utilized synthetic polymer. HPAM and its derivatives (including HAPs and AMPS-copolymers) function by increasing hydrodynamic volume through polymer chain uncoiling mechanisms in aqueous solutions [26,27,28,29,30]. Despite proven efficacy, conventional polymer flooding technologies face significant environmental challenges that limit their sustainable application. Synthetic polymer manufacturing, particularly HPAM production, relies heavily on petroleum-derived monomers, contributing substantially to the carbon footprint of EOR operations. Environmental concerns extend beyond manufacturing to include potential ecotoxicity from unreacted monomers, such as acrylamide from HPAM, as well as polymer degradation products. Produced water containing these substances requires extensive treatment before it can be disposed of or reused [8,31,32].

Additionally, conventional HPAM faces several challenges that significantly limit its performance under reservoir conditions. One of these challenges is the mechanical degradation, which occurs under high shear rates encountered during injection through wellbores and near-wellbore regions [33]. Also, chemical degradation affects HPAM performance, and it manifests primarily through hydrolysis reactions, particularly accelerated in the presence of divalent cations such as Ca^2+^ and Mg^2+^, commonly found in formation brines [34,35]. Moreover, HPAM performance is affected by thermal degradation, which becomes severe at temperatures exceeding 75–90 °C, where polymer chain scission and cross-linking reactions drastically reduce both molecular weight and viscosifying efficiency [19,36,37]. These degradation mechanisms collectively result in a substantial reduction in the solution viscosity and sweep efficiency, often rendering HPAM ineffective in reservoir environments characterized by high temperature, high salinity, and significant mechanical stress.

Such challenges and limitations have driven the petroleum industry to seek more robust alternatives, particularly focusing on naturally derived biopolymers that can withstand demanding reservoir conditions while offering environmental benefits. The limitations of conventional synthetic EOR polymers have catalyzed extensive research into “green” biopolymer alternatives characterized by renewability, biodegradability, and reduced toxicity. These materials potentially offer improved cost-effectiveness and robustness under challenging reservoir conditions while addressing environmental concerns associated with synthetic polymers.

Xanthan gum, produced through Xanthomonas campestris fermentation, represents the most successful commercial biopolymer application in EOR. Its rigid, helical structure provides superior shear, salinity, and temperature stability compared to HPAM [36,38,39,40,41]. Other established biopolymers, including welan gum, scleroglucan, schizophyllan, and guar gum [39,41,42], exhibit robust performance characteristics under reservoir conditions. However, the application of these biopolymers faces significant limitations, notably higher manufacturing costs compared to synthetic alternatives and susceptibility to microbial degradation in reservoir environments [43,44].

Green EOR materials encompass a diverse range of substances derived from natural sources, with an increasing emphasis on valorizing the agricultural waste stream. Various natural feedstocks yield EOR-active agents, primarily viscosifying polysaccharides, including starch and cellulose derivatives [39], though native forms typically require significant modification for suitable EOR properties. Fruit and vegetable waste have garnered particular attention as sustainable sources of polysaccharides. Citrus, banana, and mango peels represent valuable examples of waste-derived EOR materials [45,46]. Okra mucilage has also been investigated for EOR applications, as well as terrestrial mushrooms and extracted mucilage from hollyhock seeds [47,48,49]. These sources are attractive due to their widespread availability, low acquisition costs, and alignment with circular economic principles [40,50].

Pomegranate (*Punica granatum*) is a globally significant crop, with annual production exceeding 8 million metric tons, primarily concentrated in India, Iran, and China [51]. The fruit’s edible arils constitute only ~50% of its total weight, while the peel (exocarp and mesocarp) accounts for 30–40% (~2.4–3.2 million tons/year) and is typically discarded as waste [52]. This disposal poses environmental challenges due to the peel’s high organic load and slow decomposition, yet its biochemical composition—rich in polysaccharides (15–20%), tannins, and pectin (6–12%)—makes it an ideal candidate for valorization [53]. Pomegranate peels contain complex polysaccharide compositions, notably pectins, cellulose, and hemicellulose [54,55,56]. Pectin is a heteropolysaccharide primarily composed of α-1,4-linked D-galacturonic acid units [57], and it represents the primary functional component in pomegranate peel for EOR applications. Its efficacy stems from three critical properties: First, high molecular weight (50–300 kDa), which enables chain entanglement in aqueous solutions, increasing bulk viscosity even at low concentrations (2–12%) [58]. Secondly, carboxyl groups (-COO^−^), which promote ionic cross-linking with divalent cations (e.g., Ca^2+^ in seawater), enhancing shear resistance and stability under reservoir conditions [59,60,61]. Lastly, thermo-gelling behavior, where viscous gels would be formed at elevated temperatures during the pectin extraction from the solution [62].

Therefore, this biopolymer showed high potential and a sustainable alternative for EOR applications, leveraging abundant agricultural waste to address industry sustainability needs. Optimizing their extraction methods, particularly using saline water to mimic oilfield conditions, offers a strategic pathway to develop field-ready EOR agents. Comprehensive characterization of its rheological behavior under reservoir environments with varying salinity and temperature, and validation through core-flooding studies, would unlock its full operational viability, positioning it as a new green material for efficient oil recovery. Hence, the feasibility of using pomegranate peel waste as a novel source of biopolymer for EOR applications was examined in this study. A heat-assisted acid extraction method using synthetic seawater was used. The rheological properties of extracted biopolymer solutions were characterized under varying shear rates, temperatures, and concentrations. Then, core-flooding experiments were conducted using Berea sandstone cores of varying permeabilities, where the oil recovery performance and pressure behavior were investigated through secondary and tertiary flooding scenarios.

## 2. Materials and Methods

### 2.1. Materials

#### 2.1.1. Pomegranate Peels, Extraction Water and Brine

**Figure 2:**
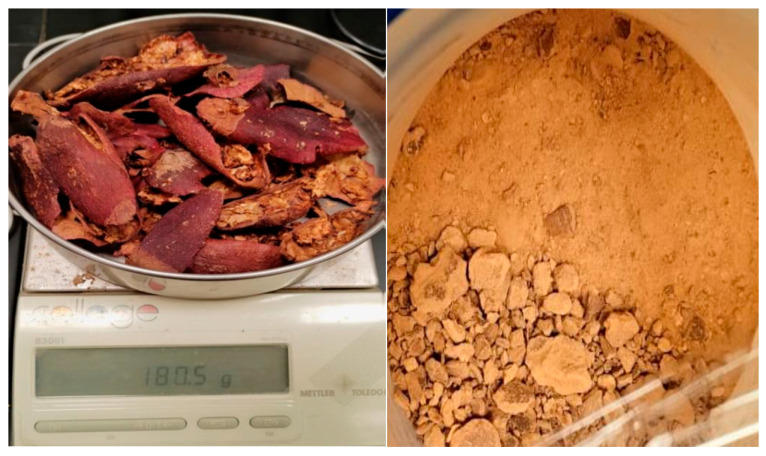
Dried and powdered pomegranate peels.

Pomegranate fruits were purchased from a local market. Peels were manually separated from the fruit pulp. The average peel mass fraction was 0.465 (peel weight/total fruit weight). Peels were oven-dried at 60 °C until a constant weight was achieved. The average dry peel mass to fresh peel mass ratio was 0.145. Dried peels were mechanically powdered to a fine consistency (Figure 2).

Synthetic formation water (FW) was prepared using different salts, as shown in Table 1. It was prepared by mixing reagent-grade salt samples with distilled water. FW of a total dissolved solids (TDS) level of 165,000 ppm was later diluted with distilled water to synthesize seawater (SW) (37,224 ppm), representing the salinity of Arab Gulf water (Table 2). At the same time, citric acid (analytical grade, >99.5%) was used to aid extraction and prepared as a 0.5% (*w*/*v*) stock solution in SW, exhibiting a pH of approximately 2.14.

#### 2.1.2. Core and Crude Oil Samples Characterization

Berea sandstone cores were used for the core-flooding experiments. Table 3 shows the petrophysical properties of the core samples. X-ray fluorescence (XRF) analysis was conducted to quantify the main elements for Berea cores (Table 4). The result showed that Berea sandstone is rich in Silica (Si), with lesser amounts of Aluminum (Al), Ferrite (Fe), and Potassium (K).

**Figure 3:**
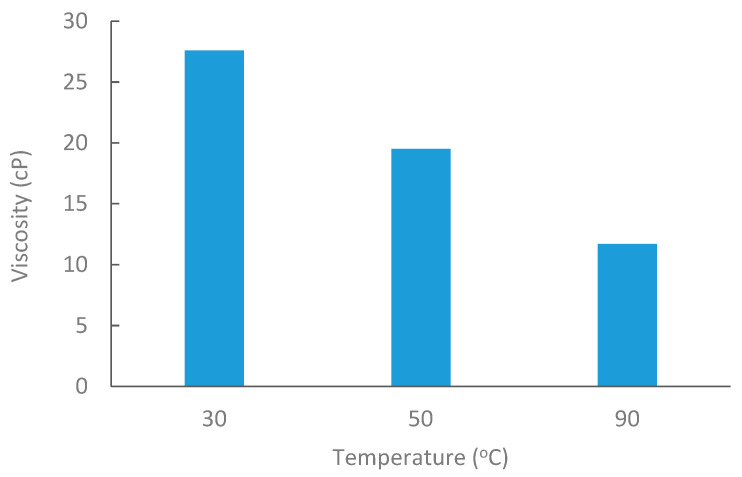
Oil viscosity at elevated temperatures @6 RPM.

Medium Saudi crude oil from Alkhafji fields was obtained with an API of 26.88. Oil viscosity was measured at 30 °C, 50 °C, and 80 °C, as shown in Figure 3. The density of crude oil was 0.89 g/cc at room temperature.

### 2.2. Experimental Setup and Equipment

#### 2.2.1. Biopolymer Extraction

**Figure 4:**
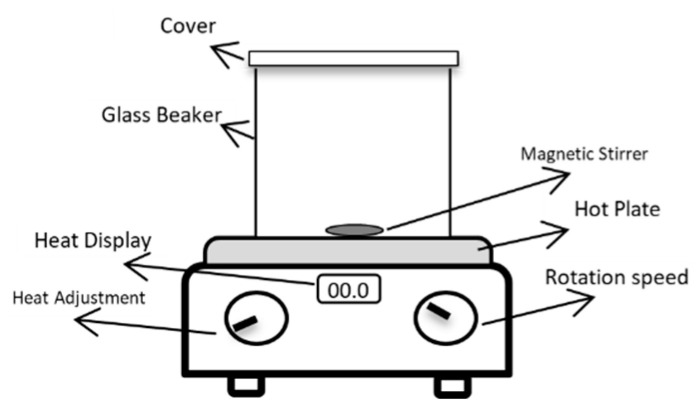
Schematic of biopolymer extraction setup. Reproduced with permission from [16], Springer Nature, 2024.

A direct hot plate heater was used for preparing the polymer solution, as shown in Figure 4. It comprises a heat source, a magnetic stirrer, and a beaker.

Fourier Transform Infrared (FTIR) and Thermogravimetric Analysis (TGA) were conducted on the extracted biopolymer to identify its morphological properties and thermal stability, where the TGA was measured over a wide range of temperatures (25–250 °C).

Also, the density of the solutions was measured using Anton Paar DMA 5000 Density Meter (Anton Paar GmbH, Graz, Austria). Additionally, the interfacial tension between oil and water, as well as between oil and a polymer solution, was measured using a KRUSS K9 tensiometer (KRÜSS GmbH, Hamburg, Germany) at ambient temperature. The viscosity of the polymer solution was measured using a Brookfield LV DV-II+P viscometer (AMETEK Brookfield Engineering, Brookfield, MA, USA) at various temperatures (25, 50, and 75 °C).

#### 2.2.2. Core-Flooding System

**Figure 5:**
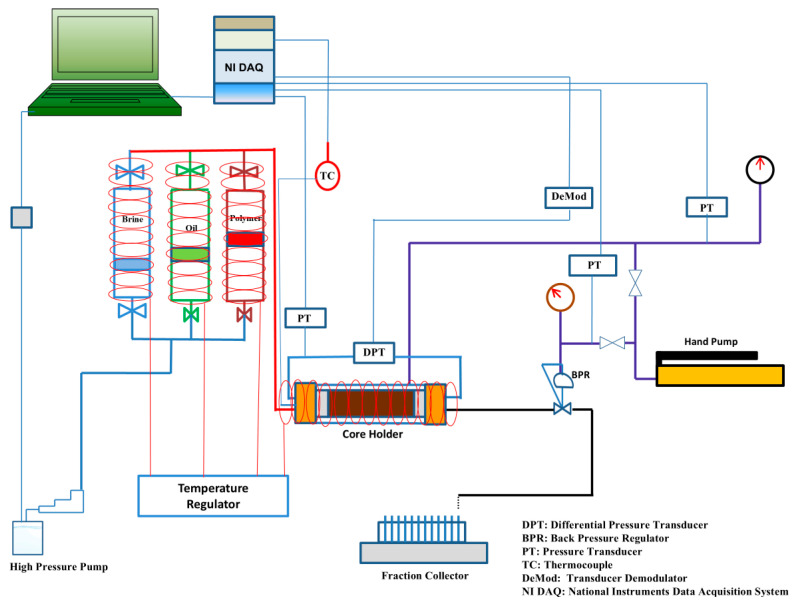
Schematic diagram of core-flooding system CFS-200.

A semi-automated core-flooding system (CFS-200) was used to conduct core-flooding experiments. The system was connected to syringe pumps for precise fluid injection and to provide confining pressure. Three floating piston accumulators were connected to the system as fluid reservoirs for FW/SW, oil, and biopolymer solution. The system was equipped with heating tapes to maintain a temperature of 75 °C throughout the experiments. Differential pressure transducers were used throughout the core to monitor the pressure drop at the inlet and outlet. In addition, a back-pressure regulator was used to maintain a constant pore pressure, and a data acquisition system was used to log pressure, temperature, and injected/produced volumes over time. A schematic diagram of the core-flooding system is shown in Figure 5.

### 2.3. Experimental Procedures

#### 2.3.1. Solution Preparation and Characterization

The extraction of the biopolymer was performed by mixing the dried pomegranate peel powder at specific concentrations (2, 5, 7, 8, 10, and 12% *w*/*v*) with seawater and 5 mL/L citric acid to achieve the target extraction environment. This mixture was continuously stirred and heated to approximately 80~90 °C for 2 h using a hot plate and magnetic stirrer (Figure 4). Following the 2 h extraction period, the solution underwent a two-stage filtration process to remove insoluble residues. Initially, the extract was filtered through cheesecloth to separate larger suspended particles of peel. Consequently, this pre-filtered solution was subjected to vacuum filtration using Whatman Grade 41 filter paper (a nominal pore size of 20–25 microns) to remove finer particulate matter. The resulting clarified biopolymer filtrate was collected for subsequent characterization and core-flooding experiments. The density and interfacial characteristics of the filtered solutions with different powdered peel concentrations (2% to 12% *w*/*v*) were then evaluated.

#### 2.3.2. Core-Flooding

The cores used in the experiment were cut to a proper length to fit in the core-flooding unit. Then, the cores were dried in the oven for 24 h at 70 °C before being saturated with FW. Dimensions and weight of the cores were recorded before saturation. Afterward, the core samples were saturated with FW using a saturation unit consisting of an air vacuum pump and a desiccator. Then, saturated weights of the cores were measured, and their pore volumes and porosity were calculated. Absolute permeability to brine (*K_w_*) was measured by flowing FW at multiple rates, applying Darcy’s Law after 100% saturation.

The core-flooding runs were started by heating the system for four hours to reach 75 °C. After reaching the designated temperature, FW was injected to establish 100% water saturation of the core, and then permeability was measured at steady-state flow using four different flow rates. Then, FW was displaced by crude oil (drainage process) at a flow rate of 0.5 cc/min. The volume of the expelled water was recorded to measure oil initial saturation (*S_oi_*), which resembles OOIP and connate water saturation (*S_wc_*). After that, FW in the accumulator was replaced with the displacing fluid (SW) and left to reach the same temperature. At this stage, the core-flooding system was ready, and flooding experiments were conducted at a constant injection rate of 0.5 cc/min, a system temperature of 75 °C, an overburden pressure of 3000 psi, and a back pressure maintained to keep the pore pressure at approximately 2000 psi. Three flooding scenarios were done as detailed in Table 5. Effluent volumes were measured to calculate recovery and water cut. Pressure readings (inlet pressure, outlet pressure, and the difference) were recorded continuously against injected Pore Volumes (PV).

The oil recovery was calculated after each core-flooding phase to assess the performance of the polymer and the best flooding scenario. Different PV of SW was used during seawater flooding (SWF) before and after water breakthrough (W_BT_) to ensure 100% water cut (W_cut_). Also, different polymer slug volumes were used during polymer flooding (PF) at different stages.

Two important factors to consider during core-flooding experiments are the resistance factor (*F_R_*) and residual resistance factor (*F_RR_*). *F_R_* is a function of the pressure drop across the core during water flooding and after polymer flooding, and *F_RR_* is the ratio of brine permeability before and after polymer flows through the core (as shown in Equations (1) and (2). *F_R_* and *F_RR_* are key parameters in assessing the performance of polymer flooding [63,64]. *F_R_* indicates the mobility reduction capability of polymer flooding, while *F_RR_* signifies the reduction in permeability resulting from polymer flooding. These values consistently exceed 1.0, leading to enhanced oil sweep efficiency. Higher *F_R_* and *F_RR_* values suggest greater potential for improving sweep efficiency and achieving increased incremental oil recovery through polymer flooding [65].

(1) $$F_{R}=\frac{{\Delta P}_{p}}{{\Delta P}_{w}}$$

(2) $$F_{RR}=\frac{k_{wi}}{k_{wa}}$$

where *F_R_* is the resistance factor, *F_RR_* is the residual resistance factor; ${\Delta P}_{p}$ is the pressure drop across the core after polymer flooding; ${\Delta P}_{w}$ is the pressure drop across the core during water flooding; *k_wi_* is brine permeability before polymer flows through the core (mD); *k_wa_* is brine permeability after polymer flows through the core (mD).

## 3. Results and Discussion

### 3.1. Biopolymer Solution

**Figure 6:**
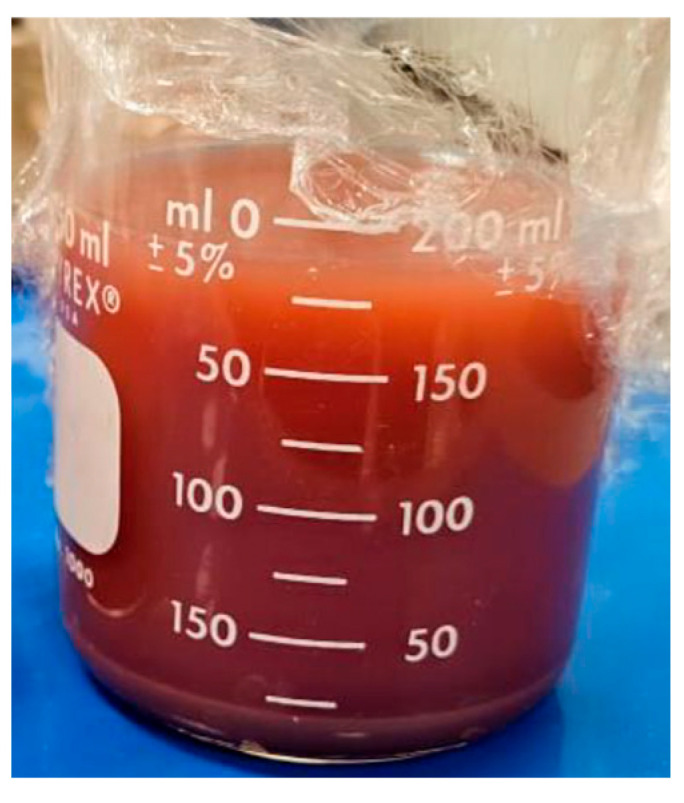
Prepared biopolymer solution.

Biopolymer solutions were successfully extracted from powdered pomegranate peels using the heat-assisted, diluted citric acid method as previously mentioned. The resulting filtrates were colored solutions (Figure 6), with the intensity of color increasing with peel concentration.

The physical state of the raw material has a significant impact on extraction efficiency. Reducing the particle size of the pomegranate peels to a powder increases the surface area available for contact with the extraction solvent [50,66]. This enhanced surface area facilitates more rapid and complete penetration of the solvent into the plant tissue, leading to improved mass transfer of the solubilized polysaccharides out of the peel matrix and into the bulk solution [67,68]. Grinding or powdering is a common pre-treatment step in polysaccharide extraction from various plant sources to maximize yield and reduce extraction time [69]. Hence, in this study, powdered pomegranate peels were used to generate biopolymer solutions, where the extraction methodology aimed to efficiently liberate the viscosifying polysaccharides from the pomegranate peel waste.

The use of an acidic medium is a cornerstone of conventional pectin and polysaccharide extraction from plant biomass [16,50]. Acid hydrolysis, typically at a pH range of 1.5 to 3.0, facilitates the breakdown of protopectin (the insoluble form of pectin in plant cell walls) into soluble pectin and other polysaccharides by cleaving the glycosidic bonds and linkages to other cell wall components like cellulose and hemicellulose [62,70]. Citric acid (pH~2.2), an organic acid, was specifically chosen over mineral acids (like HCl or H_2_SO_4_), being less corrosive, safer to handle, and yielding pectin with desirable characteristics for various applications [56,71,72]. Studies on citrus and apple pomace have demonstrated the effectiveness of pectin extraction using citric acid at similar pH ranges, often resulting in high yields and good-quality pectin [73,74]. The target pH of approximately 2.2 in this study falls within this optimal range for maximizing the solubilization of pectic substances.

A temperature range of 70–100 °C is commonly reported for acid-based pectin extraction from various fruit waste sources, including citrus peels and apple pomace [50,62,66,75]. The thermal treatment is integral to enhancing the efficiency of acid hydrolysis during polysaccharide extraction, where heating increases the kinetic energy of the system, resulting in accelerating the rate of hydrolytic reactions and improving the solubility and diffusion rate of the extracted polysaccharides from the plant matrix into the solvent. Thus, in this study, a temperature of approximately 80–90 °C and a 2 h duration were used to have a balance aimed at achieving sufficient hydrolysis and dissolution without causing excessive thermal degradation of the desired biopolymer, which can occur with prolonged exposure to very high temperatures [16,76,77]. Previous work on pomegranate peel pectin extraction also utilized similar temperature ranges, even though sometimes for food applications, confirming the suitability of this thermal regime [62].

**Figure 7:**
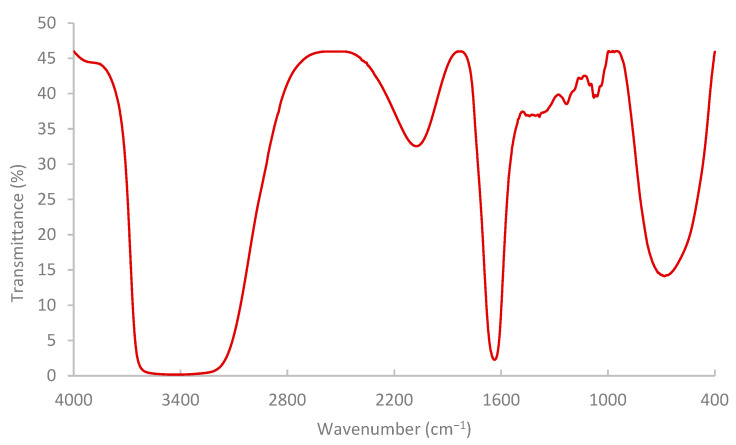
Pomegranate-peel biopolymer solution analysis by FTIR.

**Figure 8:**
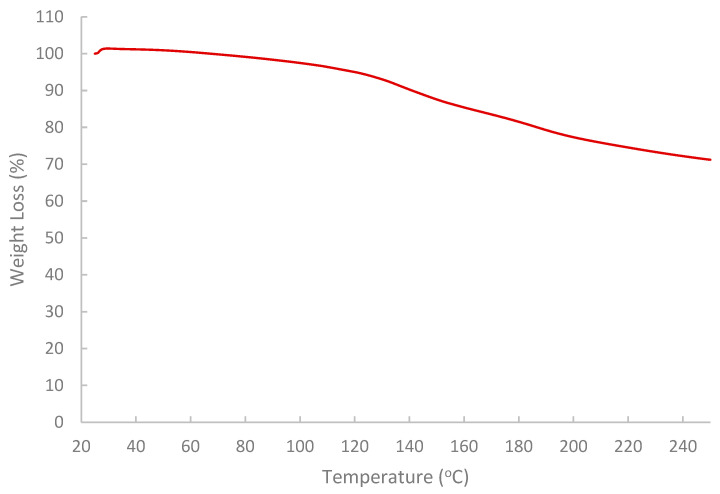
Analysis of thermal stability of the extracted biopolymer via TGA.

The results of the FTIR spectrum (Figure 7) indicated that the pomegranate-peel biopolymer exhibited a broad O–H stretching band centered near 3430 cm^−1^, aliphatic C–H stretching around 3020–2880 cm^−1^. Also, a weak shoulder was observed near 1685–1700 cm^−1^ associated with carbonyl groups (ester/acid), and strong bands at ~1637 cm^−1^ (H–O–H bending/COO^−^ asymmetric stretch) and 1442–1388 cm^−1^ (COO^−^ symmetric stretch/CH_2_ bending). The fingerprint region shows pronounced C–O–C/C–O vibrations at ~1233 and 1150–1075 cm^−1^ with features down to ~870–980 cm^−1^, confirming a polysaccharide-rich matrix (pectin/cellulose) with ionizable carboxylate groups. These functionalities are consistent with the observed shear-thinning and with viscosity sensitivity to brine composition (H-bonding and Ca^2+^/Mg^2+^ interactions), enhancing shear resistance and stability under reservoir conditions [59,60,61]. While the results of TGA (Figure 8) showed that the extracted biopolymer is thermally stable over a wide range of temperatures (25–250 °C), and supported the interpretation that the rheological behavior of the extracted biopolymer is not affected by thermal degradation at 75 °C. The tested sample retained around 99.5% mass at 75 °C with a gradual loss at 120 °C (95%). The primary decomposition zone began around 120–180 °C, where moisture or volatile components were released. At 200–250 °C, the mass remaining is near 77.3% and 71.2%, respectively.

### 3.2. Rheological Behavior of the Pomegranate Peel-Derived Biopolymer

**Figure 9:**
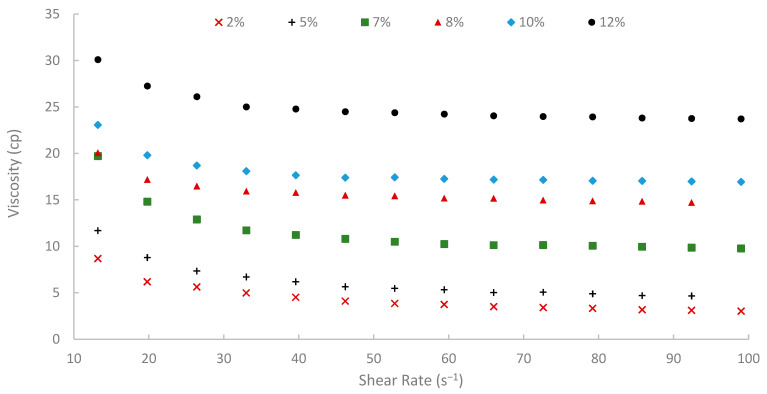
Viscosity vs. shear rate at 25 °C.

**Figure 10:**
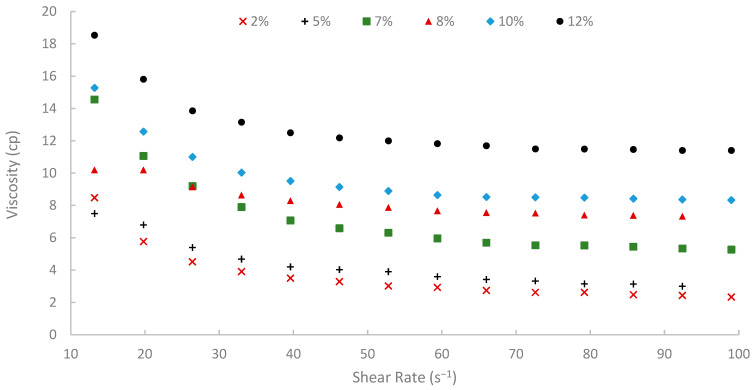
Viscosity vs. shear rate at 50 °C.

**Figure 11:**
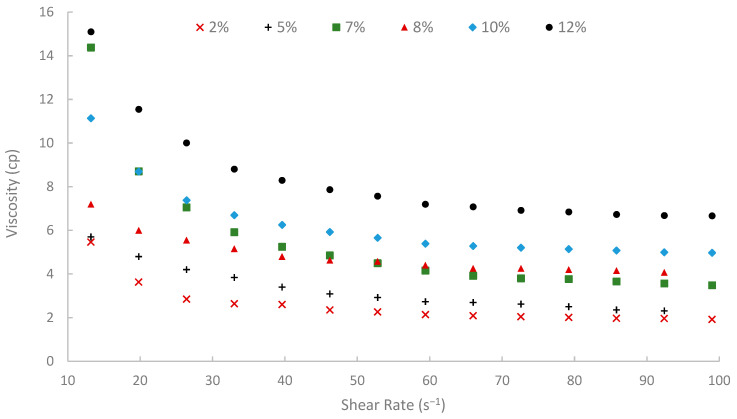
Viscosity vs. shear rate at 75 °C.

The rheological properties of biopolymer solutions were systematically examined as a function of shear rate, temperature, and peel concentration. A critical characteristic observed across all concentrations (2% to 12% *w*/*v*) and temperatures (25, 50, and 75 °C) was the pronounced shear-thinning (pseudoplastic) behavior of the pomegranate peel-derived biopolymer solutions. Viscosity decreased with increasing shear rate over the tested range of 13.20 to 99.00 s^−1^ consistently. For instance, the 7% (*w*/*v*) peel concentration solution at 25 °C showed a viscosity of 19.72 cP at 13.20 s^−1^, which progressively decreased to 9.77 cP at the higher shear rate of 99.00 s^−1^ (Figure 9). Similar shear-dependent viscosity reductions were observed for all other concentrations and temperatures, as illustrated in Figure 9, Figure 10 and Figure 11. This non-Newtonian behavior is characteristic of many polymeric solutions, including established EOR polymers such as HPAM and xanthan gum [6,7,78]. It results from the alignment of polymer chains in the direction of flow under increasing shear stress, which reduces their hydrodynamic volume and intermolecular entanglement, thereby lowering the apparent viscosity [15]. This pseudoplasticity is highly desirable for EOR applications. It allows for easier injection due to its lower viscosity at high shear rates experienced near the wellbore, while ensuring higher viscosity and thus better mobility control at the lower shear rates that prevail in the porous media [13].

Moreover, temperature displayed an inverse effect on solution viscosity, as generally predicted for polymer solutions. For any given concentration and shear rate, an increase in temperature from 25 °C to 75 °C resulted in a noticeable decrease in apparent viscosity. For example, the 10% (*w*/*v*) solution at a shear rate of approximately 72.60 s^−1^ was 17.17 cP at 25 °C, which subsequently reduced to 8.51 cP at 50 °C and further to 5.21 cP at 75 °C. This thermal thinning is typical, as increased thermal energy leads to greater molecular motion, reduced intermolecular attractive forces, and expansion of free volume, thereby lowering the resistance to flow [7,79]. Regardless of this reduction, at 75 °C (Figure 11), a representative reservoir temperature, the biopolymer solutions, particularly at higher concentrations, retained a significant portion of their viscosity (e.g., the 7% solution showed 3.80 cP at 72.60 s^−1^ and 75 °C). This suggests its potential viability for applications in high-temperature reservoirs, a condition under which conventional HPAM often suffers severe thermal and hydrolytic degradation, leading to substantial viscosity loss [19]. The ability of this waste-derived biopolymer to maintain practical viscosity levels at 75 °C is a promising finding.

The viscosity of other synthetic polymers, such as polyacrylamide (PAM) and thermo-viscosifying polymer (TVP), in the literature [80] was compared to the viscosity of the extracted biopolymer in this paper. The viscosities of PAM and TVP were calculated at different temperatures, high-salinity conditions, and at a shear rate of 10 s^−1^. When comparing the extracted biopolymer viscosity at 13.2 s^−1^ shear rate and 25 °C, the viscosities of PAM and TVP (with 0.2 wt.%) were 12.3 cP and 13.7 cP, respectively, while the viscosity of the extracted biopolymer was 19.72 cP. Also, at 75 °C, the viscosity of TVP was 11.8 cP, and the viscosity of the biopolymer was 14.38 cP. These comparisons confirm that the extracted biopolymer outperforms synthetic polymers like PAM and TVP.

**Figure 12:**
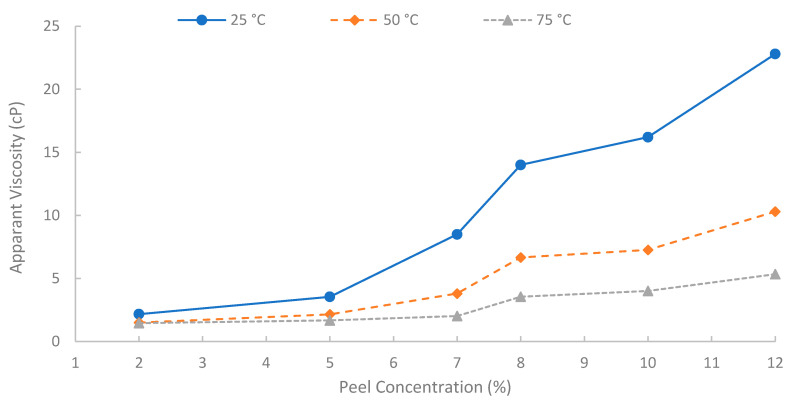
Solution viscosity at different concentrations.

A summary of apparent viscosities, derived from fitting the shear stress-shear rate data to the Bingham plastic model, is shown in Figure 12. A strong positive correlation was observed between the initial pomegranate peel concentration used for extraction and the resulting apparent viscosity of the biopolymer solutions. As peel concentration increased from 2% to 12% (*w*/*v*), the apparent viscosity increased significantly at all temperatures (25, 50, and 75 °C). This concentration-dependent viscosity enhancement is fundamental for achieving effective mobility control in EOR applications. It is attributed to the increased concentration of solubilized polysaccharide chains in the solution, leading to greater intermolecular interactions, chain entanglement, and consequently, a higher resistance to flow [6]. However, to achieve an adequate viscosity for EOR purposes while maintaining practical filterability and solution handling properties, a 7% (*w*/*v*) solution was selected for the core-flooding experiments. At 75 °C, the 7% solution showed viscosity approximately 14.3, 4.6, and 3.48 cP at shear rates of 13, 50, and 100 s^−1^, respectively, indicating favorable injectivity near the wellbore with preserved far-field viscosity. It is worth noting that the 12% solution yielded the highest viscosities, but extremely high concentrations can introduce challenges with insoluble or excessive viscosity during preparation and injection. Additionally, it is worth mentioning that when optimizing the pomegranate-peel concentration, the experiment showed that solutions of pomegranate-peel that have a high concentration (≥12%) experienced insoluble or excessive viscosity during preparation at 90 °C, and a dark color of the solution appeared, which would result in an injectivity issue. Using high pomegranate-peel concentration (≥12%) and temperature more than 75 °C would degrade the performance of the extracted biopolymer. Nevertheless, further investigation into this point should be made.

The collective rheological profile of the pomegranate peel-derived biopolymer, especially the 7% (*w*/*v*) solution, is encouraging for EOR. Its ability to generate viscosities that are significantly higher than typical injection brine (approximately 0.3–0.5 cP at reservoir temperatures) and its pronounced shear-thinning behavior propose a strong potential for improving the mobility ratio in oil displacement processes [9]. The shear-thinning characteristic is particularly advantageous, facilitating easier injection at the wellbore while allowing the polymer to exhibit higher, more effective viscosity within the lower shear environment of the reservoir matrix. The laboratory findings of this study on the rheological properties provided a solid foundation for proceeding with core-flooding experiments and evaluating oil displacement capabilities and injectivity.

### 3.3. Interfacial Properties and Their EOR Implications

Beyond the rheological properties, the interfacial characteristics of the biopolymer solutions provide further understanding of their potential EOR mechanisms. The surface tension (SFT) of the prepared biopolymer solutions against air and the interfacial tension (IFT) between the biopolymer solutions and the used oil were measured, as shown in Table 6.

The SFT values for the biopolymer solutions were found to range from approximately 49.55 mN/m to 58.8 mN/m. While these values indicate a reduction compared to pure water (~72 mN/m at 25 °C), they are not characteristic of effective surfactant systems, which typically lower SFT to 25–35 mN/m or even lower [81]. This suggests that while the extracted components possess some surface activity, they do not behave as strong surface-active agents at the air-water interface.

More critically for EOR, the effective chemical EOR processes aim to mobilize capillary-trapped oil through IFT reduction by accomplishing ultra-low IFTs (often below 0.01 mN/m). In this study, the IFT between the biopolymer solutions and the oil phase ranged from 11.0 mN/m to 13.6 mN/m, and the typical IFT of crude oil and SW was 21.8 mN/m. The measured IFT values for the pomegranate peel biopolymer solutions represented about a 50% reduction in IFT value compared to the IFT of crude oil and SW. However, these values of IFTs are considered substantially high compared to the required IFTs to significantly reduce capillary forces and trigger oil bank formation through mechanisms dependent on IFT lowering [6,15,79]. Furthermore, analysis of the SFT and IFT data across the different biopolymer concentrations (2% to 12% *w*/*v*) did not reveal a consistent, monotonic reduction with increasing concentration. Such a trend, notably a sharp decrease followed by a plateau, would typically indicate the formation of micelles and the reaching of a Critical Micelle Concentration (CMC), which is characteristic of conventional surfactants [3]. The absence of this behavior further supports the interpretation presented in this study, where the pomegranate peel biopolymer solutions primarily do not function as an IFT-reducing surfactant.

These findings on interfacial properties strongly reinforce the conclusions drawn from the rheological analysis. The developed pomegranate peel-derived biopolymer is anticipated to contribute to enhanced oil recovery primarily through the mobility control mechanism. This is achieved through the significant increase in the aqueous phase viscosity and the favorable shear-thinning behavior discussed previously, rather than through a substantial reduction in interfacial tension. It is worth mentioning that polysaccharides, such as pectins (a major component of the extract), are recognized for their emulsifying capabilities, often by providing steric stabilization rather than drastic interfacial tension reduction [62,82]. Any such effects in this system are likely to be secondary to the primary impact of viscosity modification on sweep efficiency.

### 3.4. Analysis of Core-Flooding, Recovery Performance and Pressure Profiles

The EOR potential of the pomegranate peel-derived biopolymer was rigorously evaluated through a series of core-flooding experiments. Berea sandstone cores of varying permeabilities under simulated reservoir conditions (75 °C, 165,000 ppm TDS brine, ~2000 psi pore pressure) were used. Three different flooding scenarios were investigated: a baseline seawater flood (SWF), a secondary polymer flood (PF), and a tertiary polymer flood (Table 5). The core-flooding experiments yielded distinct oil recovery and pressure responses for each injection scenario. The differential pressure data recorded during the core-flooding experiments provided crucial insights into the in situ behavior of the pomegranate peel biopolymer and its interaction with the porous medium, particularly concerning mobility control and injectivity.

**Figure 13:**
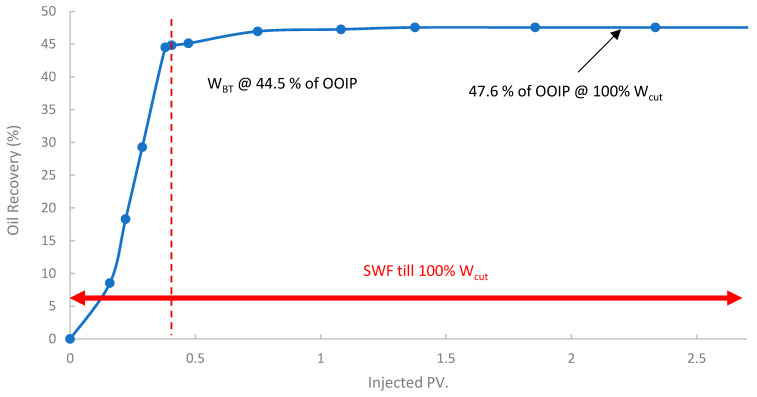
Oil recovery for 1st run—Sample B10.

**Figure 14:**
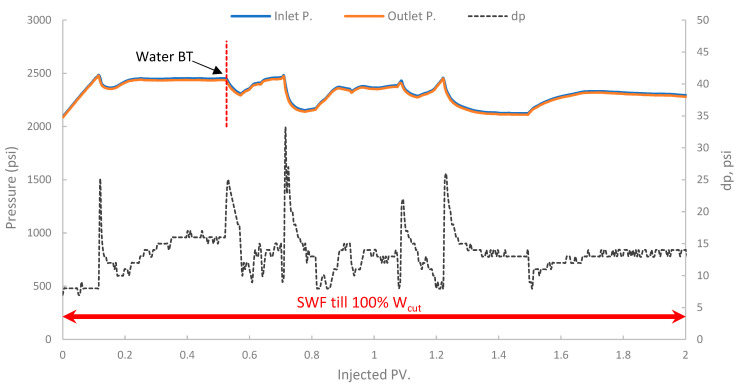
Pressure profile for 1st run—Sample B10.

In the first run, conventional SWF was conducted in a relatively high-permeability Berea core (B10, 160 mD). The results revealed that Water breakthrough (W_BT_) occurred relatively early, at an oil recovery of approximately 44.5% of the OOIP after about 0.38 PV of seawater injected, as shown in Figure 13. Continued seawater injection beyond breakthrough yielded only marginal additional oil, resulting in an ultimate oil recovery of 47.6% OOIP after approximately 1.4 PV of injected seawater. It is worth noting that no further oil was produced, even up to 2.7 PV. This level of recovery is typical for waterflooding in water-wet or intermediate-wet Berea sandstone, where significant residual oil remains due to capillary trapping and bypassing [38,83]. The differential pressure across the core during this SWF, demonstrated in Figure 14, stabilized at a consistently low value of approximately 6–8 psi after the initial oil displacement, indicating good injectivity of seawater in this high-permeability core.

**Figure 15:**
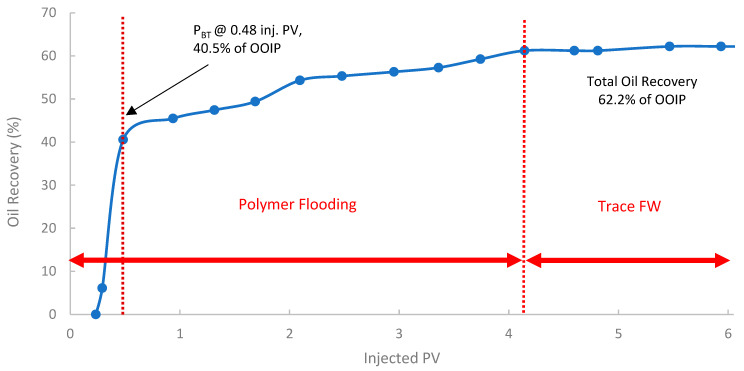
Oil recovery for 2nd run—Sample B250.

The second run evaluated the performance of the pomegranate peel biopolymer when applied in a secondary recovery mode into core B250 (118 mD). In Figure 15, the oil recovery curve shows that polymer breakthrough (P_BT_) occurred after injecting 0.48 PV at an oil recovery of approximately 40.5% of OOIP. The oil recovery reached 62.6% of OOIP after injecting 4.1 PV of biopolymer, followed by trace seawater. This represents a significant incremental oil recovery of 15% of the OOIP compared to the baseline SWF in the first run. Compared to the SWF case, W_BT_ occurred at 44.5% OOIP after ~0.38 PV injected, while in the secondary PF, P_BT_ was observed at 40.5% OOIP after ~0.48 PV. This slightly lower PV at breakthrough for the polymer flood is indicative of a more efficient, stabilized front due to the higher viscosity of the polymer solution. Such improvement is often attributed to the early establishment of favorable mobility control, which prevents premature viscous fingering and improves sweep from the outset [38,84].

**Figure 16:**
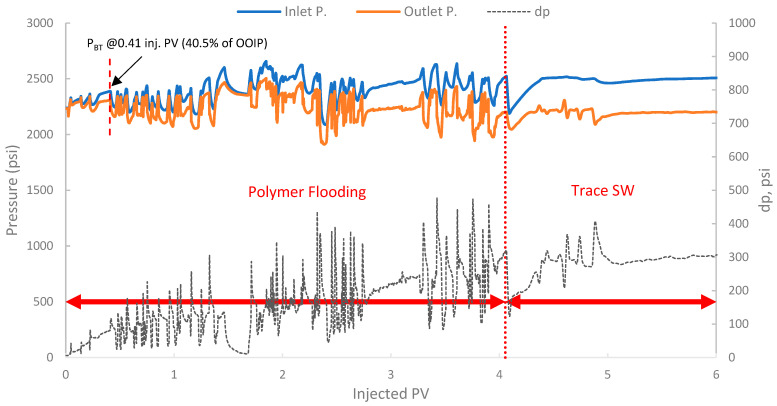
Pressure profile for 2nd run—Sample B250.

The pressure profile for this run is presented in Figure 16. It exhibited a significant and immediate increase in differential pressure upon initiation of polymer injection compared to typical waterflood injection pressures. In fact, the differential pressure reached values that fluctuated significantly, often exceeding 80–100 psi and showing an increasing trend. This behavior is indicative of the polymer’s in situ viscosifying effect but also indicates potential injectivity challenges.

**Figure 17:**
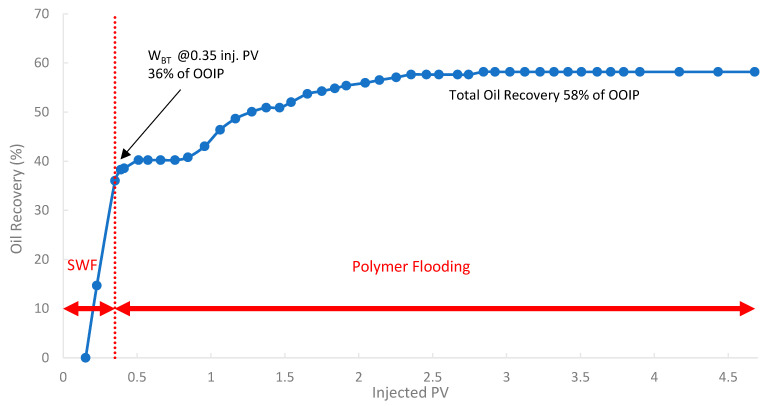
Oil recovery for 3rd run—Sample B501.

In the third run, the biopolymer was evaluated in a tertiary mode using core B501 (58 mD). The core was first subjected to SWF until WBT, which occurred at an oil recovery of 36% of OOIP after approximately 0.35 PV of injected fluid, as shown in Figure 17. Compared to the first run (core sample B10 with 160 mD), the oil recovery is lower by 8.5% due to potentially higher capillary end effects or different initial oil saturations in tighter rock formations [78]. After reaching the W_BT_ of SWF injection, subsequent injection of the biopolymer solution was conducted to mobilize additional oil. The total oil recovery was 58.0% of OOIP, where an incremental oil recovery was found to be 10.4% of the OOIP compared to SWF (run 1).

**Figure 18:**
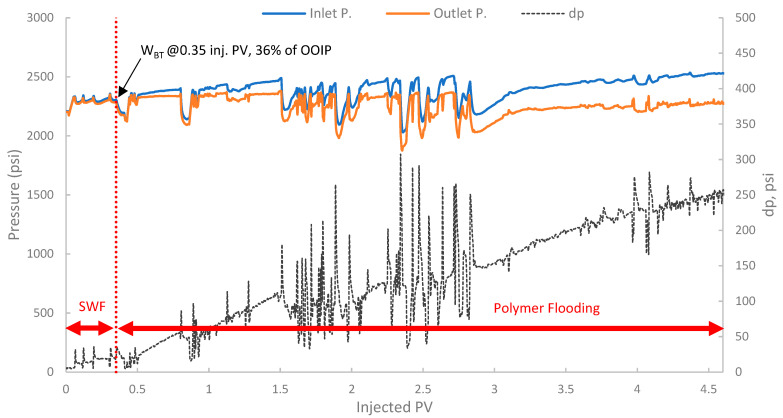
Pressure profile for 3rd run—Sample B501.

Tertiary polymer floods aim to recover oil bypassed by the preceding waterflood primarily by improving sweep efficiency, and in some cases, by altering residual oil saturation if viscoelastic effects are significant. The pressure profile in Figure 18 showed an initial stable differential pressure of around 18–20 psi during the SWF phase. Upon changing to polymer injection, a marked and sustained increase in differential pressure was observed with values exceeding 100 psi, exhibiting significant fluctuations and indicating both effective mobility control and considerable injectivity concerns.

A summary of the key oil recovery results from these three experimental runs is presented in Table 7. Both polymer flooding (PF) scenarios (2nd run: secondary PF, and 3rd run: tertiary PF) yielded substantially higher ultimate oil recovery compared to the baseline SWF (1st run). The secondary PF resulted in an incremental recovery of 14.6%, while the tertiary PF yielded an incremental 10.4% of OOIP compared to the baseline SWF. These significant incremental recovery values confirm the biopolymer’s ability to mobilize oil that was trapped or bypassed by conventional waterflooding. It is worth noting that the levels of incremental oil recovery in this study are promising and fall within the range reported for successful laboratory polymer floods using both conventional synthetic polymers and other biopolymers in sandstone cores of similar permeability [85,86].

The results indicated that the secondary polymer flood yielded a higher ultimate oil recovery (62.2%) than the tertiary polymer flood (58.0%). This observation aligns with the literature, which suggests that initiating polymer injection earlier in the life of a flood (secondary mode) can be more effective by contacting mobile oil before it becomes extensively trapped and establishing better conformance from the outset [38]. However, it is crucial to note that the third run was conducted in the core with the lowest permeability (B501, 58 mD), compared to the second run (B250, 118 mD). Lower-permeability formations inherently present greater challenges for polymer propagation. Additionally, lower permeable formations exhibit higher residual oil saturations after waterflooding, making them more susceptible to polymer retention and injectivity issues [87]. Thus, the lower permeability of the core in the third run likely contributed to its comparatively lower ultimate recovery, which may have masked the full potential of a tertiary flood if conducted in a core of similar permeability to the second run. Field studies often yield mixed results regarding the optimal timing, which is influenced by reservoir heterogeneity and economics [7].

The primary mechanism responsible for the enhanced recovery observed in both runs (2nd and 3rd runs) is improved macroscopic sweep efficiency due to mobility control. The biopolymer solution exhibited an apparent viscosity of approximately 2.02 cP (Bingham model, at 75 °C) and shear-thinning behavior. This is considerably more viscous than seawater (estimated at ~0.4 cP at 75 °C), resulting in a more favorable mobility ratio [6,7]. A lower mobility ratio suppresses viscous fingering and promotes a more piston-like displacement front, thereby contacting and displacing oil from previously unswept zones of the core [13,14]. The IFT values reported in this study (11.0–13.6 mN/m) are not sufficiently low to induce significant oil mobilization through IFT reduction, supporting the mobility control as the dominant EOR mechanism and consistent with typical polymer flooding operations.

Additionally, a substantial increase in differential pressure was observed upon the initiation of polymer injection in both runs (2 and 3), when compared to the differential pressure during the SWF injection (run one and the initial SWF phase of run 3). This marked increase directly confirms the viscosity enhancement provided by the biopolymer solution in situ and demonstrates successful mobility control [87]. The magnitude of this pressure increase relative to waterflood pressure at the same rate allows for the estimation of the *F_R_*, which quantifies the reduction in the displacing-phase mobility. The results showed that in run 1, the stabilized differential pressure was approximately 6–8 psi (Figure 14). During polymer injection in run 2 (Figure 16), the differential pressure fluctuated after an initial build-up and often exceeded approximately 100 psi before exhibiting more erratic behavior. Taking a more stable early-to-mid phase, the differential pressure of the polymer was around 100 psi (prior to severe fluctuations). However, in this case, no SWF was conducted prior to PF, and no differential pressure of water was recorded. It is worth noting that, compared to the first run, the differential pressure of water can be inferred to be in the range of 8–10 psi due to the lower permeability (118 mD) of the core used in run one (B10, 160 mD). As a result, the *F_R_* would be calculated, and it falls within the range of 10–12.5. Moreover, in run 3, the initial SWF phase provided a direct differential pressure of water of approximately 18–20 psi (Figure 18). Upon changing to polymer injection, the differential pressure of the polymer increased significantly, reaching regions of relative stability around 80–100 psi before experiencing further increases and fluctuations. As a result, *F_R_* yields values of 4.0 to 5.5. Thus, the calculated F_R_ values for both polymer flood scenarios are substantially greater than unity, confirming significant in situ viscosity development and effective mobility control by the pomegranate peel biopolymer. *F_R_* values that fall within this range are generally considered indicative of a polymer solution capable of improving volumetric sweep efficiency [7,19].

While effective mobility control was achieved, the pressure profiles during polymer injection in both runs, 2 and 3, exhibited significant fluctuations and a tendency for differential pressure to reach very high values, exceeding 100–200 psi. It is worth mentioning that this behavior, especially the sharp increases and erratic fluctuations, strongly suggests potential injectivity issues and the possibility of formation damage. Several factors in biopolymer flooding can contribute to injectivity issues, including inadequate filtration and particulate plugging, polymer aggregation or in situ gelation, and high adsorption/retention leading to permeability reduction.

Natural extracts, such as pomegranate peel biopolymer, can naturally contain suspended micro-particulates, colloidal matter, or incompletely dissolved macromolecular components, even after filtration through 20–25 micron membranes. These residual particulates can progressively plug pore throats, especially the smaller ones in tighter sections of the core or near the inlet face, leading to a rapid increase in injection pressure. This suggests that the polymer solution should be filtered to a finer level to be suitable for field applications and mitigate this risk. Additionally, while the biopolymer solution was prepared in seawater in this study, localized interactions with divalent cations (Ca^2+^, Mg^2+^ from the Berea sandstone itself) or even thermal effects over time within the core at 75 °C could potentially lead to polymer chain aggregation or the formation of microgels [54,81]. Such aggregates would be significantly larger than individual polymer coils and could readily block pore pathways, especially in the 58-mD core sample, and since pectins are known to interact strongly with divalent cations [88]. Moreover, biopolymers, especially those with charged functional groups, can exhibit significant adsorption onto rock surfaces or get mechanically entrapped in pore constrictions [89]. While Berea sandstone is relatively clean, any clay content that ranges from 3.3% to 8% as reported in [90], and the existence of elements (e.g., Aluminum (Al) and Potassium (K) in core samples), as shown in Table 4, can enhance adsorption. This progressive retention builds up a layer of polymer that reduces the effective pore diameter and permeability, leading to an increasing differential pressure [84,89]. It is worth noting that since most of the biopolymers are forms of polysaccharides and sugar, they are vulnerable to attack by environmental bacteria. Preventing such bacterial degradation can be minimized using biocides, such as formaldehyde. Under some conditions, Adsorption and insufficient mechanical stability can degrade the biopolymer. Nevertheless, biopolymers are more stable in high-salinity, high-temperature reservoirs.

## 4. Conclusions

In this study, the performance of extracting a viscosifying biopolymer from a waste material (Pomegranate peels) and its efficiency in enhancing oil recovery were experimentally investigated. Initially, biopolymer extraction was performed using a heat-assisted acid extraction method with synthetic seawater. Then, the rheological properties of the extracted biopolymer solutions were examined and optimized under varying shear rates, temperatures, and concentrations. Afterward, core-flooding experiments were conducted using Berea sandstone core samples of varying permeabilities under simulated reservoir conditions (a temperature of 75 °C, an overburden pressure of 3000 psi, and a back pressure maintained to keep the pore pressure at approximately 2000 psi). Lastly, the performance of oil recovery and pressure behavior was studied. The following are the key findings from this study:

- The results demonstrated that pomegranate peel waste as a feedstock has significant potential for producing EOR-active biopolymers capable of enhancing oil recovery under simulated reservoir conditions, offering a sustainable alternative to conventional synthetic polymers for challenging reservoirs.
- Rheological characterization of solutions derived from 2–12% (*w*/*v*) peel concentration revealed significant shear-thinning behavior, where 7% (*w*/*v*) solution was selected for EOR experiment tests. The extracted biopolymer solution at 7% (*w*/*v*) peel concentration achieved substantial incremental oil recovery values of 10.4% (SWF, then PF) to 14.6% (PF, then trace SW) using Berea sandstone core samples at 75 °C and 165,000 ppm TDS brine. The polymer flooding experiment achieved the highest ultimate recovery among the other two scenarios performed (SWF and SWF then PF), totaling 62.2% OOIP after approximately 4.1 PV of polymer injection, followed by trace seawater.
- This performance is primarily attributed to improved mobility control, evidenced by an in situ apparent viscosity of approximately 2.02 cP at 75 °C and significant resistance factors. In addition, the interfacial tension values (11.0–13.6 mN/m) were not sufficiently low to be the dominant recovery mechanism. The biopolymer solutions consistently exhibited beneficial shear-thinning (pseudoplastic) behavior across all tested concentrations and temperatures.
- While differential pressure increases confirmed effective in situ mobility control, significant injectivity challenges were observed as high and fluctuating differential pressures during polymer injection, requiring further optimization for practical field deployment. The pressure behavior analysis revealed important operational parameters for successful field implementation of the pomegranate peel biopolymer. Therefore, strategic next steps must prioritize optimizing the biopolymer solution quality and field applicability, where actionable research priorities include:
  1. Developing advanced purification protocols for the pomegranate peel extract to mitigate particulate-induced plugging and improve injectivity.
  2. Conducting detailed chemical characterization and potential modification of the biopolymer to enhance its stability and reduce adverse rock-polymer interactions.
  3. Performing extended core-flood studies incorporating refined polymer solutions to rigorously assess long-term injectivity and optimize slug design for varied permeability formations and rock types.

## Figures and Tables

**Table 1:** Synthetic FW composition.

Salt	Concentration (ppm)
NaCl	128,799
MgCl_2_	7408.5
CaCl_2_	28,312.3
NaHCO_3_	821

**Table 2:** Composition of used FW and SW.

Salt	Formation Water (FW)	Synthetic Seawater (SW)
Na^+^	51,059	11,488
Cl^−^	101,625	22,865
Ca^2+^	10,212	2297
Mg^2+^	1889	425
HCO_3_^−^	595	148.75
**TDS (ppm)**	**165,380**	**37,224**

**Table 3:** Details of the used cores in core-flooding experiments.

Formation	Core ID	Length (cm)	Pore Volume	Porosity (%)	Permeability (mD)
Berea	B10	12.3	27.11	19.3	160
Berea	B250	12.8	30.8	21.1	118
Berea	B501	10.17	20.9	18.0	58

**Table 4:** Cores XRF Quantification results.

Element	Mass %	Element	Mass %
Si	81.62211	Mn	0.131708
Al	7.986469	Cu	0.070053
Ti	0.560141	Ga	0.019511
Ca	0.34208	As	0.034362
Fe	6.196279	Cl	0.019945
S	0.01973	Sr	0.019568
Zn	0.042785	Zr	0.116792
K	2.818464	**Total**	**100**

**Table 5:** Core-flooding scenarios.

Run	Core ID	Phase 1	Phase 2
1 (Base Case)	B10	SW injection until 100% W_cut_	---
2	B250	PF	Trace SW
3	B501	SW injection until W_BT_	PF

**Table 6:** SFT and IFT measurements for biopolymer solution at different concentrations.

Peel Concentration (%)	SFT (mN/m)	IFT (mN/m)
2	58.80	12.8
5	52.43	13.6
7	57.10	11
8	49.55	11.5
10	51.47	13.3
12	49.60	11.2

**Table 7:** Summary of oil recovery results from core-flooding experiments.

Run	Scenario	Oil Recovery at BT (%)	Total Oil Recovery (%)	Incremental Oil (%)
1	100% SWF (Base Case)	44.5 (WBT)	47.6	-
2	PF, then Trace SW	40.5 (PBT)	62.2	14.6
3	SWF until WBT, then PF	36.0 (WBT pre-PF)	58.0	10.4

## Data Availability

All data supporting the findings of this work are presented within the article. For any further inquiries, please contact the corresponding author.
